# Motivational Pathways Diverge Between Frequent and Problematic Pornography Use

**DOI:** 10.1080/19317611.2025.2605517

**Published:** 2025-12-26

**Authors:** Edit Csányi, Martina D. Veszprémi, András N. Zsidó, Norbert Meskó

**Affiliations:** Institue of Psychology, University of Pécs, Pécs, EU, Hungary

**Keywords:** Pornography use motivations, problematic pornography use, sexual regulation, frequent pornography use, structural equation modeling

## Abstract

**Objectives:**

With the increasing accessibility of online sexual content, pornography consumption has become a prevalent aspect of contemporary sexual behavior. However, frequent use does not inherently indicate problematic involvement. This study aimed to clarify the motivational pathways that differentiate frequent from problematic pornography use and to examine their associations with sexual regulation and sexual motivations.

**Methods:**

A total of 890 Hungarian adults (600 women and 290 men by sex assigned at birth) completed an online survey. Regarding gender identity, most participants identified as women (64.3%) or men (34.7%), with a small proportion identifying as non-binary (0.7%) or choosing not to disclose (0.3%). Participants ranged in age from 18 to 64 years (M = 33.95, SD = 10.14). The survey assessed motivations for pornography use (PUMS), problematic pornography use (PPCS-6), sexual regulatory tendencies (SSFS), and sexual motivations (YSEX?-15). We used generalized linear modeling (GLM) and structural equation modeling (SEM) to test a theory-driven model examining how positive and negative motivations predicted pornography use patterns and sexual regulation outcomes.

**Results:**

Positive motivations—such as enhancement and partner-related use—were associated with frequent but non-problematic pornography use and with adaptive sexual regulation. In contrast, negative motivations—such as coping and emotional avoidance—were strongly linked to problematic use, marked by sexual system hyperactivation, emotion regulation difficulties, and impaired relational functioning. Only PPCS-6, not frequency of use, mediated the association between negative motivations and sexual system deactivation.

**Conclusions:**

Frequent and problematic pornography use reflect distinct psychological profiles rather than points along a severity continuum. These findings support motivation-based clinical frameworks that distinguish between functional and dysfunctional pornography use when assessing sexual health risks and designing therapeutic interventions.

## 1. Introduction

Pornography use has become a pervasive and culturally normalized phenomenon, with most adults reporting some form of engagement (Grubbs et al., 2019a). Yet despite its ubiquity, pornography use is not a unitary behavior, but a complex psychological process shaped by individual motivations and regulatory mechanisms. Understanding why people use pornography—and how these motives relate to adaptive or maladaptive outcomes—requires a theoretical framework that integrates motivation, behavioral expression, and sexual regulation.

Recent research highlights the multifaceted nature of pornography-use motivations, which range from self-enhancing and approach-oriented motives to avoidant or negatively valenced reasons (Bőthe et al., 2021a; Miller et al., 2019). These are commonly categorized as positive motivations—such as enhancing sexual pleasure, exploring fantasies, or deepening intimacy—and negative motivations, including stress reduction, emotional escape, or avoidance of discomfort. This distinction is not merely descriptive but carries important implications for psychological outcomes. Positive motivations tend to predict intentional, self-regulated use, whereas negative motivations are linked to emotion dysregulation and a higher likelihood of Problematic Pornography Use (PPU; Lewczuk et al., 2020; Meskó & Őry, 2023). Importantly, pornography use is often driven by mixed motives, such as simultaneously seeking pleasure and emotional relief (Carvalho et al., 2015), reflecting that motivational ambivalence is a central and psychologically meaningful feature of use.

Although pornography-use frequency has been a major focus of prior research, empirical evidence increasingly demonstrates that frequency alone is a poor indicator of dysfunction. Individuals may use pornography frequently for exploratory or pleasure-oriented reasons without experiencing distress or loss of control (Bőthe et al., 2020; Gola et al., 2016). By contrast, PPU reflects dysregulated sexual behavior characterized by compulsivity, distress, and impaired control over use. Conceptually, PPU emerges when pornography functions as a maladaptive emotion-regulatory strategy—used to escape negative affect or avoid interpersonal intimacy—thereby reinforcing avoidance-based coping (Grubbs et al., 2019a). From this perspective, pornography use can be viewed as a behavioral manifestation of broader sexual regulation processes, in which motivational and emotional mechanisms determine whether use remains adaptive or becomes problematic.

Understanding these processes more fully requires considering the potential mediating role of frequent pornography use (FPU), which may transmit the effects of underlying motivations onto sexual regulation outcomes. Empirical findings suggest that FPU and PPU represent distinct psychological pathways rather than points along a single continuum of dysfunction (Bőthe et al., 2020). Furthermore, recent structural validation studies (Koós et al., 2025) indicate that individuals differ systematically in their motivational profiles, supporting the plausibility of indirect pathways linking motivations, pornography-use behaviors, and regulatory outcomes. Although these studies did not directly test mediation, their findings strengthen the conceptual foundation for viewing pornography use as a potential mechanism connecting motivational patterns to sexual regulation.

Sexual regulation refers to the capacity to initiate, sustain, and modulate sexual desire and intimacy in accordance with one’s goals, values, and emotional state. When regulation is adaptive, sexual motivation supports personal agency, intimacy, and psychological well-being. When dysregulated, the same system may produce hyperactivation (e.g., excessive preoccupation with sexual activity, compulsive use) or deactivation (e.g., avoidance, emotional disengagement, or diminished desire; Bőthe et al., 2020). Pornography use motivated by approach-oriented reasons can facilitate adaptive regulation, whereas avoidance-based or compulsive motives may undermine it—leading to emotional distancing or relational strain (Ahmadzadeh et al., 2025; Garside & Goldberg, 2023; Graziani & Chivers, 2024). Thus, pornography use provides a valuable window into the mechanisms of sexual regulation, capturing both goal-directed and dysregulated expressions of sexual motivation.

Sexual motivation is one of the fundamental organizing systems of human sexuality, channeling attention, desire, and behavior toward sexually relevant goals. Classic functional models (Cooper et al., 1998) demonstrated that sexual behavior serves diverse psychological functions extending beyond reproduction—such as pleasure seeking, intimacy enhancement, self-affirmation, and stress reduction. In this framework, sex operates as a regulatory act through which individuals manage affect, relationships, and self-concept. Meston and Buss (2007) expanded this understanding by documenting hundreds of distinct sexual motives, emphasizing that sexual motivation integrates biological drives with social, cognitive, and affective functions. Some motives are approach-oriented (e.g., seeking pleasure, affection), while others are avoidance-oriented (e.g., relieving loneliness or guilt), aligning with broader emotion-regulation distinctions.

Functional models have long emphasized that sexual behavior serves diverse affective and regulatory purposes beyond reproduction (Cooper et al., 1998; Meston & Buss, 2007). From this perspective, sexual activity is one of the most potent emotion-regulation strategies in human life—it can reduce tension, enhance intimacy, and restore psychological balance. However, when sexual motivation primarily serves to manage negative effects, it may also contribute to maladaptive coping, avoidance, or dependence. Recent theoretical and empirical work (e.g., Meskó et al., 2022) supports this duality, showing that sex-related motives frequently intertwine with affect-regulatory functions. Thus, sexual motivation can be understood as a flexible system that both reflects and shapes individual differences in emotion regulation, providing a critical context for understanding why some forms of pornography use remain adaptive while others become dysregulated.

Empirical findings linking pornography use to sexual regulation remain mixed. Some studies report that pornography facilitates sexual exploration and satisfaction when used for curiosity, fantasy, or shared enjoyment (Bőthe et al., 2018; Wright & Štulhofer, 2019), whereas others associate PPU with diminished desire, relational conflict, or emotional disengagement (Ashton et al., 2020; Grubbs et al., 2019a, 2019b; Maas et al., 2018). These inconsistencies suggest that the consequences of pornography use are not determined by quantity but by qualitative differences in motivation and regulation. Pornography may thus serve as either a constructive or disruptive element of sexual regulation, depending on whether it functions as an approach- or avoidance-based coping strategy.

The present study builds upon this theoretical integration of motivation and regulation. We investigated whether positive and negative motivations for pornography use are differentially associated with distinct forms of sexual motivation (personal goal attainment, relational motives, and sex as coping) and sexual regulatory difficulties (specifically, sexual deactivation). Furthermore, we tested whether these associations were mediated by frequent pornography use (FPU) and problematic pornography use (PPU). By situating pornography use within the broader framework of sexual motivation and regulation, the study aims to clarify how motivational patterns contribute to both adaptive and maladaptive pathways of sexual behavior. This approach emphasizes that the psychological meaning of pornography use depends less on its frequency and more on its function within an individual’s sexual regulatory system. While motivations and outcomes of pornography use can vary across age and gender (Bőthe et al., 2020; Grubbs et al., 2019a, 2019b), the present study focused on identifying general psychological mechanisms linking motivational patterns and use behaviors, irrespective of demographic variation. Age- and gender-specific differences were therefore beyond the scope of this work but remain important directions for future research.

## 2. Research objectives and hypotheses

The present study adopts a motivation-centered, model-based approach to examine how positive and negative pornography use motivations relate to two behavioral indicators—frequency of pornography use and problematic pornography use—and how these pathways connect to sexual regulatory tendencies. Specifically, we aimed to identify motivational profiles associated with healthy versus maladaptive patterns of pornography use and sexual engagement.

While both the motivation for pornography use and general sexual motivation assess motivational constructs, they operate at distinct levels of behavioral specificity. Motivations for pornography use represent context-dependent regulatory tendencies within the broader system of sexual motivation. Therefore, examining their associations allows us to identify how domain-specific motives for pornography consumption relate to more general patterns of sexual motivation and regulation.

**Figure 1.**
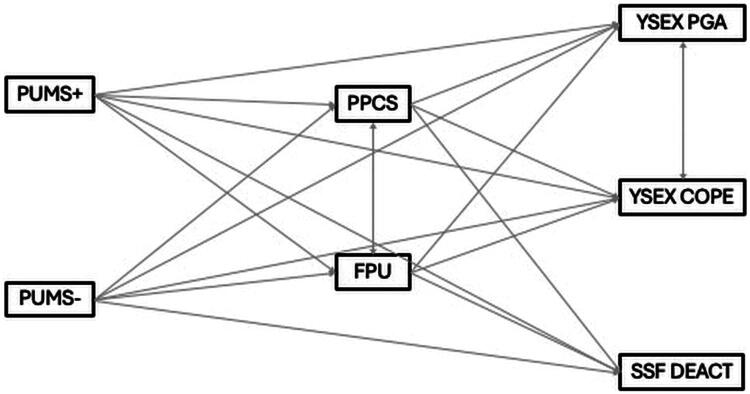
Hypothetical model of distinct psychological pathways from pornography use motivations to sexual regulatory outcomes. The model proposes that positive and negative motivations for pornography use (PUMS Positive and Negative) are associated with sexual motivations (YSEX?-15H Personal Goal Attainment, Sex as Coping) and sexual system deactivation (SSFS Deactivation) through different use-related mechanisms, including the frequency of pornography use (FPU) and problematic use tendencies (PPCS-6). Solid lines represent theoretically assumed associations. *Note*. YSEX?-15H = Hungarian Brief Version of Reasons for Having Sex Questionnaire; SSFS = Sexual System Functioning Scale; PPCS-6 = Problematic Pornography Consumption Scale – Short Form; FPU = Frequency of Pornography Use; PUMS = Pornography Use Motivations Scale.

To capture the complexity of these associations, we developed a structural equation model (SEM) specifying both direct and indirect pathways from pornography use motivations to sexual outcomes. The hypothesized structure of these motivational pathways is summarized in Figure 1. Pornography use frequency (FPU) and problematic pornography use (PPCS) were included as potential mediators. As a preparatory step, we conducted a series of general linear models (GLMs) to identify empirically relevant predictors and outcomes that informed the model specification.

Based on theoretical considerations and the results of these preliminary analyses, we formulated the following hypotheses:

- **H1**. Positive motivations for pornography use will be positively associated with personal goal-oriented and relational sexual motivations, whereas negative motivations will be negatively associated with personal goal-oriented sexual motivation.
- **H2**. Negative motivations for pornography use will be positively associated with sexual deactivation, whereas positive motivations will be negatively associated with sexual deactivation.
- **H3**. Positive and negative motivations for pornography use will be indirectly associated with sexual deactivation and sexual motivation through pornography use frequency and problematic pornography use.

## 3. Method

### 3.1. Participants

A total of 890 Hungarian adults (600 women and 290 men by sex assigned at birth) completed the survey. The sample consisted of adults aged 18 to 64 years. Given this gender distribution and the study’s focus on psychological mechanisms rather than group comparisons, gender differences were not examined statistically. Demographic characteristics of the sample are summarized in Table 1.

**Table 1.** Demographic characteristics of the sample (N = 890).

Variable	Category	n	%
Sex assigned at birth	Women	600	67.4
	Men	290	32.6
Gender identity	Woman	572	64.3
	Man	309	34.7
	Non-binary	6	0.7
	Prefer not to say	3	0.3
Education	Primary	13	1.5
	Secondary	418	47.0
	Tertiary	459	51.6
Employment status	Full-time	444	49.9
	Part-time	97	10.9
	Self-employed	99	11.1
	Student	211	23.7
	Unemployed	28	3.1
	Retired	10	1.1
Place of residence	Small village	43	4.8
	Large village/small town	104	11.7
	City	302	33.9
	County capital	165	18.5
	Capital/metropolitan area	276	31.0
Relationship status	Single, no sexual partner	144	16.2
	Single, occasional partners	94	10.6
	In a committed relationship (no cohabitation)	193	21.7
	In a committed cohabiting relationship	200	22.5
	Married/registered partnership	254	28.5
	Other	5	0.6
Sexual orientation	Heterosexual	738	82.9
	Bisexual	70	7.9
	Heteroflexible/bicurious	34	3.8
	Gay	14	1.6
	Lesbian	11	1.2
	Demisexual	9	1.0
	Pansexual	7	0.8
	Asexual	6	0.7
	Other	1	0.1
Number of lifetime sexual partners	0	68	7.5
	1	111	12.5
	2	84	9.4
	3	72	8.1
	4	43	4.8
	5–6	106	11.9
	7–9	87	9.8
	10–19	113	12.7
	≥ 20	136	15.3

*Note*. Percentages may not total 100% due to rounding.

Data were collected via an anonymous online survey distributed in Hungarian. The survey link was shared through the university’s mailing list and posted by psychology students on their personal social media pages accompanied by a brief study invitation. Participation was voluntary, uncompensated, and open to all adults aged 18 years or older.

Participants without prior sexual experience (n = 68, 7.5%) were retained in the analyses, as the study focused on general psychological mechanisms of sexual motivation and regulation rather than on behavioral sexual activity.

To ensure that our sample size was sufficient for the planned statistical analyses, we conducted a priori power analyses using two approaches. For the general linear model (GLM) analysis, we used the pwr package in R and adopted a conservative estimation (effect size f = 0.05, power = 0.95, α = 0.05, degrees of freedom = 4). This calculation yielded a minimum required sample size of n = 371.

For the structural equation modeling (SEM), we used the web interface of the semPower package (Moshagen & Bader, 2024), assuming a conservative model fit criterion (RMSEA = 0.05, power = 0.95, α = 0.05), which indicated a required sample size of n = 616.

Our final sample of 890 participants therefore exceeded both thresholds, ensuring sufficient power for both the GLM and SEM analyses.

### 3.2. Procedure

Data were collected via an anonymous online survey administered through SurveyMonkey. The survey link was disseminated through Facebook and other Hungarian social networking platforms to recruit a heterogeneous sample. Participation was voluntary and uncompensated. Before beginning the questionnaire, participants provided informed consent and were assured of the confidentiality and anonymity of their responses.

The study protocol was approved by the United Ethical Review Committee for Research in Psychology (reference number: 2023-111). The research fully adhered to international ethical standards, including the Declaration of Helsinki (Rickham, 1964) and the Ethical Principles of Psychologists and Code of Conduct of the American Psychological Association (APA, 2002).

### 3.3. Materials

#### 3.3.1. Pornography Use Motivations Scale (PUMS)

Pornography use motivations were assessed using the 24-item Pornography Use Motivations Scale (PUMS; Bőthe et al., 2021a; Hungarian adaptation: Koós et al., 2025). The PUMS captures a broad range of self-reported reasons for pornography consumption and has been validated across multiple cultures and languages, including Hungarian. Respondents who reported intentional pornography use in the past year were asked to indicate how often each motivation applied to them on a 7-point Likert scale (1 = never, 7 = always). The scale comprises eight factors: Sexual Pleasure, Sexual Curiosity, Emotional Distraction, Stress Reduction, Fantasy, Boredom Avoidance, Lack of Sexual Satisfaction, and Self-Exploration. In the present study, we used two higher-order dimensions: Adaptive motivations (e.g., “I watch porn to arouse myself sexually”) and Maladaptive motivations (e.g., “I watch porn to distract myself from negative feelings”). Each subscale demonstrated excellent internal consistency (McDonald’s ω = .84–.91 in the current study). The PUMS provides a multidimensional assessment of both adaptive and maladaptive motivations, enabling fine-grained modeling of the quality of pornography use.

#### 3.3.2. Frequency of pornography use

The frequency of online pornography consumption over the past year was assessed with a single item rated on a 10-point Likert-type scale ranging from 0 (never) to 9 (at least once a day). Higher scores indicate more frequent pornography use.

#### 3.3.3. Problematic Pornography Consumption Scale – Short Form (PPCS-6)

Problematic pornography use was assessed with the 6-item short version of the Problematic Pornography Consumption Scale (PPCS-6; Bőthe et al., 2021b). This brief instrument captures core indicators of problematic use, including salience, mood modification, tolerance, withdrawal, conflict, and relapse. Participants were asked to recall their pornography use over the past six months and rate each item on a 7-point Likert-type scale ranging from 1 (never) to 7 (all the time), with higher scores indicating greater severity. A sample item is: “I feel unable to control my pornography use,” which reflects the relapse dimension. A total score of 20 or higher is recommended as a cutoff for identifying potentially problematic use. In the present study, the PPCS‑6 showed good internal consistency (McDonald’s ω = .85).

#### 3.3.4. Sexual System Functioning Scale (SSFS)

The dynamics of the sexual system were assessed using the 24-item Sexual System Functioning Scale (SSFS; Birnbaum et al., 2014; Hungarian adaptation by Meskó & Őry, 2023). Items are rated on a 7-point Likert-type scale ranging from 1 (not at all) to 7 (very much), reflecting general agreement with statements about sexual emotions, cognitions, and behaviors. The SSFS captures two theoretically grounded dimensions of sexual regulation: Sexual Hyperactivation (e.g., “I am constantly preoccupied with sexual thoughts”) and Sexual Deactivation (e.g., “I distance myself from sexuality even in an intimate relationship”), which parallel anxious and avoidant patterns within attachment theory. Although these dimensions are related to aspects of sexual regulation and experience, the scale does not directly assess physiological or clinical aspects of sexuality but rather motivational and self-regulatory tendencies within the sexual domain. In the present sample, both subscales demonstrated good internal consistency (Hyperactivation: McDonald’s ω = .89; Deactivation: ω = .86).

#### 3.3.5. Reasons for Having Sex Questionnaire – Hungarian Short Form (YSEX?-15H)

General sexual motivation was measured using the 15-item Hungarian short form of the Reasons for Having Sex Questionnaire (YSEX?-15H; Meskó et al., 2022). The scale was developed and validated in a large Hungarian sample and captures universal motives for sexual intercourse. Participants rated the extent to which each reason applied to their past sexual experiences on a 5-point Likert-type scale ranging from 1 (none of my sexual experiences) to 5 (all of my sexual experiences). The instrument comprises three subscales: Personal Goal Attainment (5 items; ω = .73), Relational Reasons (5 items; ω = .79), and Sex as Coping (5 items; ω = .76). Sample items include: “I had sex because it felt good” (Personal Goal Attainment), “I had sex to express love for my partner” (Relational Reasons), and “I had sex to forget about my problems” (Sex as Coping). Higher scores reflect greater endorsement of the corresponding motivational domain. Importantly, the YSEX?-15H allows respondents without prior sexual experience to report the likelihood that each reason would apply to them in future sexual situations, ensuring validity for sexually inexperienced participants as well.

### 3.4. Data analysis

We first conducted five general linear models (GLMs) to test which of the following predictors—Frequency of Pornography Use, PPCS-6, PUMS Positive, and PUMS Negative—were significantly associated with the three subscales of the YSEX?-15H (i.e., Personal Goal Attainment, Relational Reasons, and Sex as Coping) and the two subscales of the SSFS (i.e., Hyperactivation and Deactivation). Each GLM included one YSEX?-15H or SSFS subscale as the dependent variable and the four predictor variables mentioned above as independent variables.

Next, we ran two additional GLMs to examine whether PUMS Positive and PUMS Negative were significant predictors of the PPCS-6 and Frequency of Pornography Use, which served as the dependent variables in these models.

Based on the results of the GLMs, we then specified and tested a structural equation model (SEM) to evaluate the hypothesized relationships among pornography use motivations, behavioral indicators, and sexual regulatory outcomes. In the SEM, the outcome variables were YSEX?-15H Personal Goal Attainment, YSEX?-15H Sex as Coping, and SSFS Deactivation. The remaining subscales of the YSEX?-15H and SSFS were not included in the SEM because they did not show significant associations with PPCS-6 or Frequency of Pornography Use in the previous analyses.

In the model, Personal Goal Attainment was predicted by PUMS Positive, PPCS-6, and Frequency of Pornography Use; Sex as Coping was predicted by PPCS-6; and SSFS Deactivation was predicted by PUMS Negative and Frequency of Pornography Use. On the next level, PPCS-6 and Frequency of Pornography Use were each predicted by both PUMS Positive and PUMS Negative. We also allowed covariances between PPCS-6 and Frequency of Pornography Use, and between the two YSEX?-15H subscales, based on theoretical considerations and prior empirical correlations. All variables were standardized before being entered into the model.

The SEM was estimated using the Maximum Likelihood method. Model fit was evaluated according to widely accepted criteria (Browne & Cudeck, 1992; Hu & Bentler, 1998): a relative chi-square (χ^2^/df) of 3 or less; Comparative Fit Index (CFI) and Tucker-Lewis Index (TLI) values of 0.95 or higher; and Root Mean Square Error of Approximation (RMSEA) and Standardized Root Mean Square Residual (SRMR) values of 0.08 or less.

The anonymized dataset is openly available at the following address: https://osf.io/86csm/?view_only=e68725f22cbf4d2b8f31753ed5a561ab. All statistical analyses were performed using the JAMOVI statistical program, version 2.5 for Windows (Jamovi Project, 2024). A complete correlation matrix of all study variables is presented in Supplementary Table 1.

## 4. Results

General linear models (GLMs) are summarized in Table 2. Overall, pornography-use motivations showed distinct association patterns across sexual motivation, sexual regulation, and behavioral engagement. Approach-oriented motives (i.e., positive motives such as pleasure seeking, curiosity, or intimacy; Positive Pornography Use Motivation Scale – PUMS Positive) were positively related to personal goal–oriented sexual motivation and relational reasons (from the Why Have Sex?–15H), as well as to the frequency of pornography use (FPU). In contrast, avoidance-based motives (i.e., negative motives such as stress reduction or emotional escape; Negative Pornography Use Motivation Scale – PUMS Negative) were positively related to sex-as-coping motivation and to problematic pornography use (PPU; Problematic Pornography Consumption Scale–6 – PPCS-6).

**Table 2.** Results of the general linear models.

				95% CI					
	Variable	B	SE	Lower	Upper	β	df	t	*p*
**YSEX?-15H Personal Goal Attainment**	(Intercept)	10.052	0.111	9.833	10.270	−0.000	885	90.195	<.001
	PUMS Positive	0.052	0.011	0.030	0.075	0.216	885	4.544	<.001
	PUMS Negative	−0.030	0.015	−0.058	−0.001	−0.107	885	−2.039	0.042
	PPCS.SUM	0.125	0.033	0.061	0.190	0.183	885	3.813	<.001
	FPU	0.159	0.072	0.017	0.302	0.104	885	2.199	0.028
**YSEX?-15H Relational Reasons**	(Intercept)	13.800	0.147	13.512	14.088	−0.000	885	93.945	<.001
	PUMS Positive	0.061	0.015	0.031	0.090	0.198	885	4.001	<.001
	PUMS Negative	−0.016	0.019	−0.054	0.022	−0.045	885	−0.831	0.406
	PPCS.SUM	0.043	0.043	−0.042	0.129	0.050	885	1.001	0.317
	FPU	0.057	0.096	−0.130	0.245	0.030	885	0.599	0.549
**YSEX?-15H Sex as Coping**	(Intercept)	8.528	0.111	8.309	8.747	−0.000	885	76.561	<.001
	PUMS Positive	0.022	0.011	−0.000	0.045	0.098	885	1.953	0.051
	PUMS Negative	0.015	0.015	−0.013	0.044	0.058	885	1.050	0.294
	PPCS.SUM	0.086	0.033	0.021	0.151	0.132	885	2.616	0.009
	FPU	−0.161	0.072	−0.304	−0.019	−0.111	885	−2.229	0.026
**SSFS Hyperactivation**	(Intercept)	27.248	0.407	26.450	28.046	−0.000	885	67.008	<.001
	PUMS Positive	0.092	0.042	0.010	0.175	0.107	885	2.204	0.028
	PUMS Negative	0.205	0.053	0.101	0.310	0.206	885	3.853	<.001
	PPCS.SUM	0.204	0.120	−0.031	0.440	0.084	885	1.703	0.089
	FPU	−0.484	0.264	−1.003	0.035	−0.089	885	−1.831	0.067
**SSFS Deactivation**	(Intercept)	27.831	0.385	27.077	28.586	−0.000	885	72.354	<.001
	PUMS Positive	−0.051	0.040	−0.129	0.026	−0.064	885	−1.297	0.195
	PUMS Negative	0.157	0.050	0.058	0.256	0.170	885	3.118	0.002
	PPCS.SUM	0.108	0.114	−0.115	0.331	0.048	885	0.954	0.340
	FPU	−1.380	0.250	−1.871	−0.889	−0.273	885	−5.518	<.001
**PPCS-6**	(Intercept)	9.283	0.119	9.049	9.517	−0.000	887	77.903	<.001
	PUMS Positive	0.039	0.011	0.017	0.061	0.110	887	3.538	<.001
	PUMS Negative	0.265	0.013	0.240	0.290	0.649	887	20.832	<.001
**FPU**	(Intercept)	0.000	0.023	−0.046	0.046	0.000	887	0.000	1.000
	PUMS Positive	0.032	0.002	0.027	0.036	0.465	887	14.740	<.001
	PUMS Negative	0.025	0.002	0.020	0.030	0.320	887	10.127	<.001

The associations of YSEX?-15H and SSFS subscales with Adaptive and Maladaptive PUMS, PPCS and FPU; and the associations of PPCS and FPU with Adaptive and Maladaptive PUMS.

*Note.* YSEX?-15H = Hungarian Brief Version of Reasons for Having Sex Questionnaire; SSFS = Sexual System Functioning Scale; PPCS-6 = Problematic Pornography Consumption Scale – Short Form; FPU = Frequency of Pornography Use; PUMS = Pornography Use Motivations Scale.

Personal goal–oriented sexual motivation was positively associated with the frequency of pornography use, problematic pornography use, and positive motives, and negatively associated with negative motives. Sex-as-coping motivation was positively associated with both the frequency of pornography use and problematic pornography use. Relational reasons were positively associated only with positive motives and were therefore excluded from the SEM.

Regarding sexual regulation outcomes, sexual deactivation (emotional withdrawal or reduced engagement; from the Sexual System Functioning Scale, SSFS) was negatively associated with the frequency of pornography use and positively associated with negative motives, whereas sexual hyperactivation (preoccupation with sexual activity) was positively associated with both positive and negative motives. Because hyperactivation showed no association with behavioral indicators (i.e., frequency and problematic pornography use), it was not included in the SEM.

Finally, both problematic pornography use and the frequency of pornography use were positively predicted by both positive and negative motives, indicating that greater motivational intensity—whether approach- or avoidance-based—is linked to higher levels of pornography use and to problematic involvement. Although the explained variance in some GLM models was modest, this pattern is typical for complex motivational frameworks in which multiple overlapping processes contribute to individual differences. Crucially, variance inflation factor (VIF) diagnostics indicated no substantial multicollinearity among predictors, supporting the interpretation that the observed associations reflect theoretically meaningful—albeit partial—effects.

**Figure 2.**
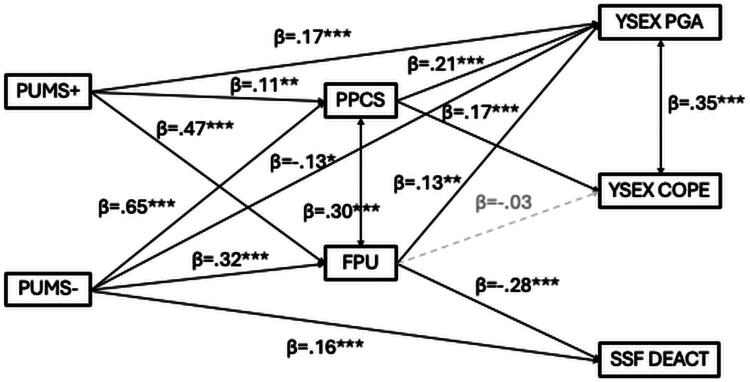
Structural equation model testing the potential mediating roles of problematic pornography use (PPCS-6) and frequency of pornography use (FPU) in the associations between pornography use motivations (PUMS Positive and Negative) and sexual regulatory outcomes. All pathways identified in preliminary GLMs are included. Statistically significant pathways are shown in black (*p < .05, **p < .01, ***p < .001); non-significant paths are shown in grey. All estimates are standardized. *Note*. YSEX?-15H = Hungarian Brief Version of Reasons for Having Sex Questionnaire; SSFS = Sexual System Functioning Scale; PPCS-6 = Problematic Pornography Consumption Scale – Short Form; FPU = Frequency of Pornography Use; PUMS = Pornography Use Motivations Scale.

Next, we tested whether the frequency of pornography use and problematic pornography use function as differential mediators between pornography-use motivations and sexual regulatory outcomes. Only GLM-identified predictors were retained in the structural equation model (SEM). The model showed good fit, χ^2^(4) = 12.1, p = .017, CFI = .995, TLI = .977, RMSEA = .048, 90% CI [.018, .080], SRMR = .015 (see Figure 2 and Supplementary Table 2 for full details). In the SEM, personal goal–oriented sexual motivation (R^2^ = .12) was positively predicted by problematic pornography use, the frequency of pornography use, and positive motives, and negatively predicted by negative motives. Sex-as-coping motivation (R^2^ = .02) was positively predicted by problematic pornography use but not by the frequency of pornography use. Sexual deactivation (R^2^ = .05) was negatively predicted by the frequency of pornography use and positively predicted by negative motives. Both problematic pornography use (R^2^ = .51) and the frequency of pornography use (R^2^ = .52) were positively predicted by both positive and negative motives.

In addition, the covariance between problematic pornography use and the frequency of pornography use, and the covariance between personal goal–oriented and coping-related sexual motives, were positive and statistically significant, indicating theoretically coherent interrelations among these constructs.

## 5. Discussion

### 5.1. Motivational pathways to pornography use and sexual regulation

The present study examined motivational pathways linking pornography use to sexual regulatory outcomes, emphasizing the distinct roles of approach- and avoidance-based motives. Both the GLM and SEM analyses revealed that positive motivations for pornography use—such as curiosity, pleasure seeking, and fantasy exploration—were primarily associated with adaptive forms of sexual motivation, particularly goal-oriented and relational motives. In contrast, negative motivations—such as distress reduction or emotional distraction—were linked to maladaptive regulatory outcomes, including sexual deactivation and problematic pornography use.

These findings are consistent with prior research emphasizing motivational heterogeneity as a determinant of the psychological effects of pornography use (Bőthe et al., 2021a; Miller et al., 2019). Positive, approach-oriented motives tend to promote agentic and intimacy-focused sexual behavior (Cooper et al., 1998; Meston & Buss, 2007), whereas avoidance-based motives reflect attempts to regulate negative affect through external stimulation. The latter have been consistently associated with emotional dysregulation, compulsivity, and reduced engagement in partnered sexual contexts (Grubbs et al., 2019a; Kor et al., 2014).

This pattern aligns with emotion regulation models (Aldao & Nolen-Hoeksema, 2010), suggesting that distress-driven pornography use may provide temporary relief but ultimately reinforce maladaptive coping. It is important to note, however, that using sex to cope with emotions is not inherently maladaptive. In certain contexts, sexual activity can serve as an adaptive form of emotion regulation, helping to reduce tension, enhance intimacy, or promote emotional connection. The distinction lies in regulatory flexibility: coping through sex may become maladaptive when it functions primarily as avoidance or emotional suppression rather than emotional engagement (Cooper et al., 1998; Meskó et al., 2022). This nuance highlights the need for future studies and clinical assessments to differentiate between adaptive and maladaptive coping motives, as current self-report instruments may not fully capture this variability.

In contrast, the relationship between positive motivations and goal-oriented sexual motives was mediated by the frequency of pornography use, but not by problematic use, reinforcing that frequent use is not necessarily maladaptive (Gola et al., 2016). These findings underscore that motivational context, rather than mere frequency, determines whether pornography use supports or hinders sexual regulation.

Overall, the results support a motivation-informed model distinguishing adaptive and maladaptive pathways. Positive motivations may enhance intimacy and engagement, whereas avoidance-driven motives—especially when coupled with compulsive use—can undermine sexual responsiveness. Yet these categories often overlap in real life: individuals may pursue pornography both for pleasure and emotional escape, a motivational ambivalence that future studies could model more precisely using person-centered or longitudinal approaches.

### 5.2. Sexual Scripts, dysregulation, and the role of problematic pornography use

The observed link between avoidance-based motivations and sexual deactivation reflects broader mechanisms of emotion dysregulation and internalized sexual scripts. Our findings support the view that problematic pornography use is not merely behavioral excess but an indicator of maladaptive coping shaped by personal vulnerabilities and sociocultural narratives about sexuality (Bőthe et al., 2020; Grubbs et al., 2019a).

Although not directly measured in the present study, previous research suggests that pornography-related sexual scripts—culturally shared schemas about how sexual interactions should unfold—may provide a broader context for understanding our findings. Such scripts often emphasize performance, novelty, and male-centered pleasure, which can contribute to emotional detachment and dissatisfaction in partnered contexts (Wright, 2011; Wright & Tokunaga, 2018). Repeated exposure to these idealized portrayals may recalibrate sexual arousal patterns through reward-based learning processes, where high-intensity stimuli condition the brain’s reward system and diminish sensitivity to ordinary sexual cues (Baranowski et al., 2019; Gola & Draps, 2018; Hilton, 2013). This framework aligns with our interpretation that problematic pornography use reflects not only behavioral excess but also a dysregulated pattern of sexual motivation and emotion regulation (Bőthe et al., 2020; Grubbs et al., 2015).

Our data showed that problematic use—but not frequency—mediated the link between negative motivations and sexual deactivation, highlighting its distinctive psychological role. Frequent pornography use, especially when driven by curiosity or fantasy exploration, may reflect adaptive exploration; in contrast, problematic use often co-occurs with emotional disengagement and diminished sexual vitality. This distinction aligns with process-based therapeutic frameworks, including cognitive-behavioral and acceptance-based approaches, which conceptualize compulsive sexual behavior as a maladaptive emotion regulation strategy that offers short-term relief but perpetuates avoidance in the long term (Lotfi et al., 2021; Twohig & Crosby, 2010).

Moreover, problematic pornography use was positively associated with coping-related sexual motivation, suggesting that individuals who experience loss of control in solitary sexual behavior may also engage in partnered sex as a means of emotional regulation. This convergence across solitary and partnered contexts supports transdiagnostic models of compulsive sexual behavior emphasizing shared mechanisms such as impulse control deficits, experiential avoidance, and emotional inflexibility (Kafka, 2014; Reid, 2016). Importantly, our findings do not pathologize all pornography use. Instead, they underscore that its outcomes depend on motivational and regulatory context. When used for escape or distress relief, pornography may reinforce maladaptive scripts and weaken intimacy; when integrated into value-consistent, relationally attuned sexuality, it may serve adaptive or exploratory purposes.

### 5.3. Clinical implications: toward motivation-focused interventions

The current findings have important implications for the assessment and treatment of pornography-related difficulties. Rather than emphasizing behavioral metrics such as frequency, clinicians should focus on the motivational functions of pornography use (Grubbs et al., 2019b; Kraus et al., 2018). Differentiating between approach- and avoidance-oriented motives can help identify whether use reflects curiosity and exploration or distress-driven coping, thereby avoiding overpathologization while improving risk assessment.

Therapeutic frameworks such as Cognitive Behavioral Therapy (CBT), Acceptance and Commitment Therapy (ACT), and Dialectical Behavior Therapy (DBT)—each targeting emotion regulation, impulse control, and value alignment—are particularly suited to the dysregulatory patterns observed in association with problematic pornography use and sexual deactivation (Lotfi et al., 2021; Twohig & Crosby, 2010). ACT-based approaches, emphasizing psychological flexibility, may be especially useful for individuals whose use serves as experiential avoidance, by helping them tolerate emotional discomfort without resorting to compulsive behavior.

Therapeutic assessment may also benefit from sexual motivation mapping—helping clients identify their sexual goals (e.g., intimacy, pleasure, connection) and reflect on how their behaviors support or hinder these aims. This approach may be particularly valuable for those who rely on sex for coping, who could benefit from alternative emotion regulation strategies and relational skill-building. Interventions could integrate emotion-focused therapy, mindfulness, and sexual script restructuring to foster more authentic and relationally meaningful sexuality (Ahmadzadeh et al., 2025; Garside & Goldberg, 2023; Graziani & Chivers, 2024).

In summary, a motivation-focused clinical lens can enhance prevention and intervention efforts by prioritizing the why behind pornography use rather than the how often, supporting healthier sexual regulation and emotional well-being.

### 5.4. Limitations and future directions

While the present study offers novel insights into the motivational underpinnings of pornography use and their associations with sexual regulation, several limitations must be acknowledged.

First, the cross-sectional design precludes causal inference. Although structural equation modeling (SEM) permits the testing of theoretically driven directional hypotheses, the observed associations may be bidirectional or shaped by unmeasured third variables. Longitudinal and experimental research is needed to determine whether specific pornography-use motivations precede the development of problematic patterns or sexual regulatory difficulties over time (Kraus et al., 2020).

Second, all variables were assessed via self-report instruments, which are subject to response biases, including social desirability, recall inaccuracy, and introspective limitations. This concern is particularly salient in the domain of sexual behavior, where cultural taboos, privacy concerns, and personal shame may distort reporting (King, 2022; Schroder et al., 2003; Tourangeau & Yan, 2007). Future studies should consider multi-method designs, including partner reports, behavioral tasks, or ecological momentary assessment (EMA), to improve ecological validity and reduce common method variance (Shiffman et al., 2008).

Third, although we conceptually distinguished between positive and negative pornography-use motivations, these categories may not fully capture the complexity or ambivalence of real-life motives. Individuals may use pornography simultaneously for pleasure and distress regulation, and motivational states may fluctuate over time. Future research should investigate the temporal dynamics and situational moderators of motivation—such as affective states, relationship quality, or perceived stress—using more fine-grained, momentary assessment tools (Carvalho et al., 2015).

Fourth, although our sample size was adequate for the planned SEM analyses, the sample may not be representative of the broader population in terms of cultural background, sexual orientation, clinical status, or age diversity. Most participants were young adults from a WEIRD (Western, Educated, Industrialized, Rich, and Democratic) cultural context, which may limit the generalizability of the findings. Cross-cultural and lifespan studies—including clinical populations—are needed to explore how motivational patterns may differ across sociocultural and developmental contexts.

Fifth, the current model did not examine several other potentially relevant mediators or outcomes, such as body image, attachment style, relational communication, or sexual satisfaction. Including such variables in future models could provide a more comprehensive understanding of the psychological pathways linking pornography-use motivations with sexual well-being. In particular, the construct of psychological flexibility—central to ACT-based frameworks—may be a promising moderator or mediator. It could help explain why some individuals are able to maintain adaptive sexual regulation despite engaging in pornography use for emotion-regulatory reasons (Gloster et al., 2017).

Sixth, pornography-use frequency (FPU) was assessed using a single-item self-report measure. Although such measures are practical and have been widely employed in prior research (e.g., Bőthe et al., 2020), they may lack the nuance needed to capture key contextual variables, such as usage setting (e.g., solo vs. partnered), modality (e.g., video, image, live), intensity (e.g., time spent per session), and fluctuations across time or emotional states. A more comprehensive assessment—utilizing multidimensional instruments or diary-based methods—could improve construct validity and enable a more fine-grained analysis of associations with psychological outcomes (Grubbs et al., 2015).

Seventh, the sample was predominantly female (approximately two-thirds of participants), which may limit the generalizability of the results, as problematic pornography use has been found to be more prevalent among men (Grubbs et al., 2019b). Although the present study did not examine gender or age differences directly, our focus on psychological mechanisms provides a foundation for future investigations to test whether these pathways operate similarly across demographic and developmental groups.

In summary, while this study advances a motivationally informed framework for understanding the psychological correlates of pornography use, future research is needed to refine, expand, and validate this model across diverse populations, timeframes, and sociocultural contexts. Such efforts will be crucial for developing evidence-based, tailored interventions that promote adaptive sexual regulation and emotional well-being.

## 6. Conclusions

This study contributes to a more nuanced understanding of pornography use by adopting a motivational framework that distinguishes between positive and negative drivers of consumption. Our findings demonstrate that these motivational dimensions are differentially associated with sexual regulatory outcomes, both directly and indirectly, through the frequency of pornography use and problematic involvement. Specifically, positive motivations were linked to personal goal–oriented and relational sexual motives, whereas negative motivations were more strongly associated with sex-as-coping tendencies, sexual deactivation, and problematic use.

Importantly, the results underscore that frequent pornography use is not inherently maladaptive. Instead, the motivational and emotional regulation context plays a critical role in shaping outcomes. By integrating both motivational and behavioral variables into a unified structural model, this study moves beyond simplistic, frequency-based classifications and offers a differentiated perspective that aligns with emerging empirical and therapeutic frameworks emphasizing psychological flexibility and adaptive regulation.

These insights have implications not only for empirical research but also for clinical practice—particularly in identifying individual motivational profiles that may benefit from targeted interventions. Ultimately, understanding why individuals engage with pornography—rather than merely how often—represents a critical step toward supporting healthier sexual expression and psychological well-being.

## Supplementary Material

Supplementary_Materials.docx

## Data Availability

The data that support the findings of this study are available at: https://osf.io/86csm/?view_only=e68725f22cbf4d2b8f31753ed5a561ab.
