# Supplementary material for: Motivational Pathways Diverge Between Frequent and Problematic Pornography Use
Source: Int J Sex Health. 2025 Dec 26;38(2):635–49. doi: 10.1080/19317611.2025.2605517 (PMC13203051; doi:10.1080/19317611.2025.2605517)
Supplement: Supplementary_Materials.docx [file WIJS_A_2605517_SM5888.docx]

Motivational Pathways Diverge Between Frequent and Problematic Pornography Use

**Supplementary Materials**

In these Supplementary Materials, we present the correlation matrix for all the variables, and the exact statistical values for the Structural Equation Model included in our study.

**Supplementary Table 1.** Pearson correlation matrix for all variables included in our study.

| **Variable** |  | **PPCS**  **SUM** | **FPU** | **PUMS**  **Positive** | **PUMS**  **Negative** | **YSEX?-15H**  **PGA** | **YSEX?-15H**  **RR** | **YSEX?-15H**  **SAC** | **SSFS**  **Deactivation** |
| --- | --- | --- | --- | --- | --- | --- | --- | --- | --- |
| **FPU** | Pearson's r | .628 | — |  |  |  |  |  |  |
|  | p-value | <.001 | — |  |  |  |  |  |  |
|  | 95% CI Upper | .666 | — |  |  |  |  |  |  |
|  | 95% CI Lower | .587 | — |  |  |  |  |  |  |
| **PUMS**  **Positive** | Pearson's r | .548 | .681 | — |  |  |  |  |  |
|  | p-value | <.001 | <.001 | — |  |  |  |  |  |
|  | 95% CI Upper | .593 | .714 | — |  |  |  |  |  |
|  | 95% CI Lower | .501 | .644 | — |  |  |  |  |  |
| **PUMS**  **Negative** | Pearson's r | .724 | .633 | .675 | — |  |  |  |  |
|  | p-value | <.001 | <.001 | <.001 | — |  |  |  |  |
|  | 95% CI Upper | .754 | .671 | .709 | — |  |  |  |  |
|  | 95% CI Lower | .691 | .592 | .637 | — |  |  |  |  |
| **YSEX?-15H**  **PGA** | Pearson's r | .290 | .299 | .315 | .238 | — |  |  |  |
|  | p-value | <.001 | <.001 | <.001 | <.001 | — |  |  |  |
|  | 95% CI Upper | .349 | .357 | .373 | .299 | — |  |  |  |
|  | 95% CI Lower | .229 | .238 | .255 | .175 | — |  |  |  |
| **YSEX?-15H**  **RR** | Pearson's r | .145 | .168 | .216 | .144 | .496 | — |  |  |
|  | p-value | <.001 | <.001 | <.001 | <.001 | <.001 | — |  |  |
|  | 95% CI Upper | .209 | .231 | .277 | .207 | .544 | — |  |  |
|  | 95% CI Lower | .080 | .103 | .152 | .079 | .445 | — |  |  |
| **YSEX?-15H**  **SAC** | Pearson's r | .158 | .075 | .133 | .149 | .368 | .418 | — |  |
|  | p-value | <.001 | .026 | <.001 | <.001 | <.001 | <.001 | — |  |
|  | 95% CI Upper | .221 | .140 | .197 | .212 | .423 | .471 | — |  |
|  | 95% CI Lower | .093 | .009 | .068 | .084 | .310 | .362 | — |  |
| **SSFS**  **Deactivation** | Pearson's r | -.036 | -.179 | -.109 | -.012 | -.123 | -.284 | .201 | — |
|  | p-value | .284 | <.001 | .001 | .726 | <.001 | <.001 | <.001 | — |
|  | 95% CI Upper | .030 | -.115 | -.044 | .054 | -.058 | -.222 | .263 | — |
|  | 95% CI Lower | -.101 | -.242 | -.174 | -.077 | -.187 | -.343 | .137 | — |
| **SSFS**  **Hyperactivation** | Pearson's r | .236 | .167 | .231 | .282 | .069 | .042 | .310 | .341 |
|  | p-value | <.001 | <.001 | <.001 | <.001 | .038 | .207 | <.001 | <.001 |
|  | 95% CI Upper | .297 | .230 | .293 | .342 | .135 | .108 | .368 | .398 |
|  | 95% CI Lower | .172 | .102 | .168 | .221 | .004 | -.023 | .249 | .282 |

*Note*. YSEX?-15H PGA = Hungarian Brief Version of Reasons for Having Sex Questionnaire *Personal Goal Attainment*; YSEX?-15H RR = Hungarian Brief Version of Reasons for Having Sex Questionnaire *Relational Reasons*; YSEX?-15H SAC = Hungarian Brief Version of Reasons for Having Sex Questionnaire *Sex as Coping*; SSFS = Sexual System Functioning Scale; PPCS-6 = Problematic Pornography Consumption Scale – Short Form; FPU = Frequency of Pornography Use; PUMS = Pornography Use Motivations Scale.

**Supplementary *Table 2.*** *The exact statistical values including the point estimates (B), standard errors (SE), standard estimate (ß) values, z values, and p values for the structural equation model we conducted.*

|  |  |  |  | **95% CI** | |  | **95% CI** | |  |  |
| --- | --- | --- | --- | --- | --- | --- | --- | --- | --- | --- |
| **Dependent** | **Predictor** | **Estimate** | **SE** | **Lower** | **Upper** | **β** | **Lower** | **Upper** | **z** | **p** |
| FPU | PUMS Negative | 0.3195 | 0.0331 | 0.2522 | 0.38345 | 0.3195 | 0.2576 | 0.3815 | 9.653 | <.001 |
| FPU | PUMS Positive | 0.4651 | 0.0286 | 0.4124 | 0.52130 | 0.4651 | 0.4087 | 0.5214 | 16.281 | <.001 |
| PPCS-6 SUM | PUMS Negative | 0.6494 | 0.0451 | 0.5656 | 0.73917 | 0.6494 | 0.5790 | 0.7198 | 14.385 | <.001 |
| PPCS-6 SUM | PUMS Positive | 0.1103 | 0.0362 | 0.0387 | 0.18130 | 0.1103 | 0.0390 | 0.1816 | 3.050 | 0.002 |
| YSEX?-15H PGA | FPU | 0.1327 | 0.0523 | 0.0303 | 0.23814 | 0.1330 | 0.0303 | 0.2356 | 2.536 | 0.011 |
| YSEX?-15H PGA | PPCS SUM | 0.2056 | 0.0604 | 0.0787 | 0.32375 | 0.2060 | 0.0841 | 0.3279 | 3.403 | <.001 |
| YSEX?-15H PGA | PUMS Negative | -0.1251 | 0.0575 | -0.2327 | -0.00664 | -0.1253 | -0.2391 | -0.0116 | -2.174 | 0.030 |
| YSEX?-15H PGA | PUMS Positive | 0.1707 | 0.0461 | 0.0752 | 0.25772 | 0.1711 | 0.0802 | 0.2619 | 3.701 | <.001 |
| YSEX?-15H SAC | FPU | -0.0345 | 0.0413 | -0.1101 | 0.05071 | -0.0345 | -0.1154 | 0.0463 | -0.835 | 0.404 |
| YSEX?-15H SAC | PPCS SUM | 0.1739 | 0.0486 | 0.0759 | 0.26086 | 0.1741 | 0.0751 | 0.2731 | 3.579 | <.001 |
| SSFS Deactivation | FPU | -0.2770 | 0.0387 | -0.3535 | -0.20397 | -0.2774 | -0.3487 | -0.2060 | -7.161 | <.001 |
| SSFS Deactivation | PUMS Negative | 0.1548 | 0.0376 | 0.0798 | 0.22992 | 0.1550 | 0.0795 | 0.2304 | 4.119 | <.001 |
| FPU | PPCS SUM | 0.1416 | 0.0198 | 0.103 | 0.1844 | 0.2983 | 0.229 | 0.3675 | 7.14 | <.001 |
| YSEX?-15H PGA | YSEX SAC | 0.3214 | 0.0356 | 0.251 | 0.3939 | 0.3486 | 0.282 | 0.4148 | 9.02 | <.001 |

*Note*. YSEX?-15H PGA = Hungarian Brief Version of Reasons for Having Sex Questionnaire Personal Goal Attainment; YSEX?-15H SAC = Hungarian Brief Version of Reasons for Having Sex Questionnaire Sex as Coping; SSFS = Sexual System Functioning Scale; PPCS-6 = Problematic Pornography Consumption Scale – Short Form; FPU = Frequency of Pornography Use; PUMS = Pornography Use Motivations Scale.
